# Probing the active site tryptophan of *Staphylococcus aureus* thioredoxin with an analog

**DOI:** 10.1093/nar/gkv1255

**Published:** 2015-11-17

**Authors:** Markus Englert, Akiyoshi Nakamura, Yane-Shih Wang, Daniel Eiler, Dieter Söll, Li-Tao Guo

**Affiliations:** 1 Department of Molecular Biophysics & Biochemistry, Yale University, New Haven, CT 06520-8114, USA; 2 Department of Chemistry, Yale University, New Haven, CT 06520-8114, USA

## Abstract

Genetically encoded non-canonical amino acids are powerful tools of protein research and engineering; in particular they allow substitution of individual chemical groups or atoms in a protein of interest. One such amino acid is the tryptophan (Trp) analog 3-benzothienyl-l-alanine (Bta) with an imino-to-sulfur substitution in the five-membered ring. Unlike Trp, Bta is not capable of forming a hydrogen bond, but preserves other properties of a Trp residue. Here we present a pyrrolysyl-tRNA synthetase-derived, engineered enzyme BtaRS that enables efficient and site-specific Bta incorporation into proteins of interest *in vivo*. Furthermore, we report a 2.1 Å-resolution crystal structure of a BtaRS•Bta complex to show how BtaRS discriminates Bta from canonical amino acids, including Trp. To show utility in protein mutagenesis, we used BtaRS to introduce Bta to replace the Trp28 residue in the active site of *Staphylococcus aureus* thioredoxin. This experiment showed that not the hydrogen bond between residues Trp28 and Asp58, but the bulky aromatic side chain of Trp28 is important for active site maintenance. Collectively, our study provides a new and robust tool for checking the function of Trp in proteins.

## INTRODUCTION

**Figure 1.**
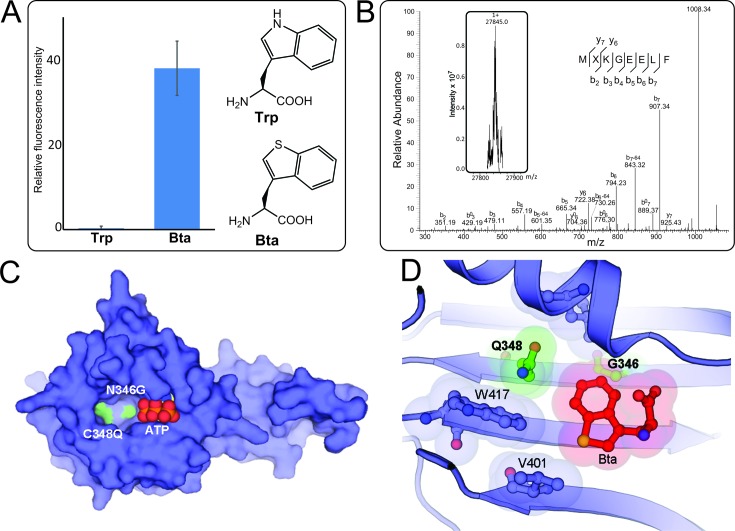
The BtaRS evolved from PylRS bearing Asn346Gly and Cys348Gln mutations discriminates Bta from Trp. (**A**) Suppression of the UAG codon at position 2 of sfGFP-2UAG mRNA supported by BtaRS in the presence of Bta or Trp was measured by fluorescence intensity. (**B**) Molecular weight determination of the protein sfGFP-Bta and its MXKGEELF (X denotes Bta) fragment. The deconvoluted singly charged ESI-MS spectrum of sfGFP-Bta by FT-ICR MS (inserted Fig. and see Supplementary Figure S4 for the full MS image) and tandem mass spectra of the MXKGEELF (X denotes Bta) fragment from full-length sfGFP-Bta protein are shown. The full-length sfGFP-Bta protein was expressed using the BtaRS•tRNA^Pyl^_CUA_ pair in the presence of 1 mM Bta. Calculated molecular weight of full-length sfGFP-Bta protein is 27 845 Da ([M+H]^1+^); we found 27 845 Da. *b*^0^ or *y*^0^ stands for *b*-H_2_O or *y*-H_2_O. (**C**) The overall structure of BtaRS in the absence of the Bta shows the positions of Asn346Gly and Cys348Gln mutations in the active site pocket. The side chains of Asn346Gly/Cys348Gln double mutations are shown in green, and the bound ATP is in combination of red and orange. (**D**) The anatomy of the BtaRS active site illustrates the substrate recognition. Mutations from the parent PylRS are indicated in bold, and the mutated side chains are shown in green. The shadows indicate the van der Waals radius of Bta and the surrounding residues that are involved in substrate recognition.

Trp is one of the 20 canonical amino acids, which plays unique roles in maintaining protein folds and promoting protein–protein and protein–ligand interactions, because of the various types of non-covalent interactions formed by its large hydrophobic yet polar aromatic side chain (1–5). Trp is also the main source of intrinsic fluorescence in most proteins (6). Collectively, these properties make Trp a useful probe for protein studies, and an attractive target for protein engineering and design (7,8). In this context, numerous Trp analogs have been incorporated into proteins to endow them with new qualities (9), to explore the Trp contribution to protein folding and function (10), and to reveal the effects of chemical group substitutions (7,11). For instance, the Trp analog 3-benzothienyl-l-alanine (Bta) (Figure 1A) was used to selectively abolish Trp's ability to form hydrogen bonds (10).

Genetic code expansion studies to incorporate non-canonical amino acids (ncAAs) require an orthogonal aminoacyl-tRNA synthetase (aaRS)•tRNA pair that will form the ncAA-tRNA whose anticodon recognizes a UAG or UGA codon in the mRNA for the desired protein (12). For Bta two systems have been used to date: a mutated yeast phenylalanyl-tRNA synthetase•tRNA^Phe^_CUA_ pair (13) and an optimized *S. cerevisiae* tryptophanyl-tRNA synthetase•tRNA^Trp^_CUA_ (14,15).

The pyrrolysyl-tRNA synthetase (PylRS)•tRNA^Pyl^ pair has proven itself an outstanding system for genetic code expansion. The large amino acid binding site of PylRS (16,17) enabled the selection of over 100 ncAAs, and it is the only pair whose orthogonality and expression in both bacterial and eukaryotic systems are well established (18,19). Here, when we attempted to evolve a PylRS variant for efficient 3-iodo-L-phenylalanine (3-I-Phe) incorporation, we unexpectedly obtained a variant that efficiently incorporates Bta. This allowed the structure elucidation of a BtaRS•Bta complex, and incorporation of this ncAA into the active site of *Staphylococcus aureus* thioredoxin.

## MATERIALS AND METHODS

### PylRS library construction and selection, enzymatic assay and superfolder GFP screening

These experiments were carried out essentially as described previously (20). The standard positive-negative-positive selections were performed for 3-I-Phe incorporation. The chloramphenicol acetyltransferase gene harboring a TAG codon at position 112 was used as the positive selection marker. In negative selection, the translation of TAG codons at positions 13 and 44 in the toxic gene *ccdB* eliminated the PylRS variants that charge tRNAs with canonical amino acids. The sfGFP gene bearing a TAG codon at position 2 was coexpressed with tRNA^Pyl^ and PylRS mutants in the presence of ncAAs. The expression of wild-type (WT) sfGFP was used as a positive control.

### Structure determination of BtaRS in complex with Bta

As the archaeal full-length PylRS enzymes are poorly soluble, we used truncated versions (residues 185–454) of PylRS variants, as has been done in past investigations of these enzymes (16,21). The C-terminal domain (CTD, residues 185–454) of BtaRS was overexpressed and purified as described earlier (16,20). After size exclusion chromatography, the purified BtaRS was mixed with 5 mM Bta and 5 mM ATP in the buffer containing 10 mM HEPES (pH 7.4), 0.3 M NaCl, 5 mM MgCl_2_ and 1 mM DTT, and then concentrated to 12 mg/ml by using a centrifugal filter (Millipore; MWCO, 10 kDa). Crystals of BtaRS with Bta were obtained by sitting drop vapor diffusion against 100 mM HEPES–NaOH pH 7.0, 5 mM MgCl_2_ and 14% PEG3350 at 20°C. Prior to the data collection, crystals were soaked in the cryoprotectant solution containing 100 mM HEPES–Na pH 7.0, 5 mM MgCl_2_, 14% PEG3350 and 35% ethylene glycol. The 2.1 Å-resolution data set (*I*/*σ* = 2) of BtaRS complexed with Bta was collected from a single crystal, at 100 K, at the beam line 24ID-C at the Advanced Photon Source Synchrotron (Argonne, IL). The diffraction data were processed by using *XDS* (22). After a rigid body refinement with the structure of the PylRS CTD (PDB ID: 2ZIM) as a start model by *phenix.refine* (23), the structure model of BtaRS with Bta was rebuilt using *Coot* (24). The final model was refined by using *phenix.refine*. MolProbity was used to validate the model (25). Data collection and refinement statistics are summarized in Supplementary Table S1. All figures of PylRS mutants were prepared with *PyMOL* (The PyMOL Molecular Graphics System, Version 1.5.0.4, Schrödinger, LLC).

### Overexpression and purification of thioredoxin, its mutants, TrxR and ArsC

The coding sequences of *S. aureus* thioredoxin (Trx), its mutants, Trx reductase (TrxR) and arsenate reductase (ArsC) were synthesized with codons optimized for *E. coli* expression through IDT (Coralville, USA). ArsC and TrxR were cloned into pET15b vector between the NdeI and BamHI sites, resulting in pET15b-arsC and pET15b-trxR, respectively, which allowed the overexpression of the N-terminal His_6_-tagged recombinant proteins followed by Ni-NTA purification. Trx and variants were cloned into pET20b vector between the NdeI and XhoI sites, resulting in pET20b-trx, pET20b-trxW28A and pET20b- trxW28TAG, which allowed the overexpression and purification of a C-terminal His_6_-tagged protein.

pET15b-arsC, pET20b-trx, pET20b-trxW28A and pET15b-trxR were transformed into *E. coli* strain BL21(DE3). The cultures of 1-l lysogeny broth (LB) with 50 μg/ml ampicillin were inoculated 1/100 from a pre-culture and incubated at 37°C until the optical density (*A*_600_) reached 0.6. After addition of isopropyl-thiogalacoside (IPTG) to a final concentration of 0.1 mM, the temperature was shifted to 25°C for overnight (16 h) incubation. pET20b-trxW28TAG was co-transformed with the PylRS orthogonal translation system consisting of pCDF-BtaRS and pCAM-PylT into BL21(DE3). A pre-culture of 250 ml LB with the antibiotics ampicillin (50 μg/ml), spectinomycin (25 μg/ml) and tetracycline (5 μg/ml) was grown at 37°C till the OD reached 1.0. The cells were harvested by centrifugation and washed twice with the same volume of 1× M9 salts (Difco, BD, New Jersey, USA). The cells were resuspended in 250 ml GMML medium [1× M9 salts, 2 mM MgSO_4_, 0.1 mM CaCl_2_, 1% (v/v) glycerol, 0.3 mM leucine] supplemented with proper antibiotics, 1 mM IPTG, 0.1 mM Trp and 2 mM Bta (Chem-Impex, Wood Dale, USA) and incubated overnight (16 h) at 25°C.

The Ni-NTA purifications and the Trx enzymatic activity assays were performed in a tent (Coy Lab products, Grass Lake, USA) under anaerobic atmosphere (90% N_2_, 5% CO_2_, 5% H_2_). Harvested cells were resuspended in lysis buffer (20 mM HEPES–NaOH, pH 7.7, 500 mM NaCl and 10 mM imidazole) and broken by sonication. The lysate was clarified by centrifugation (20 000 g, 45 min) and applied on Ni-NTA resin for standard purification using a wash buffer with 40 mM imidazole and elution buffer with 200 mM imidazole. The protein concentrations were determined by the absorption at 280 nm (*A*_280_) using a NanoDrop (ThermoScientific, Wilmington, USA) and calculated molar extinction coefficients. SDS-PAGE analysis using 10 μg each of the eluted proteins verified their purity >95% after Coomassie Brilliant Blue staining.

The presence of Bta as the read-through product of the pET20b-trxW28TAG expression was confirmed by liquid chromatography coupled to tandem mass spectrometry (LC–MS/MS) (Supplementary Figure S1). In brief, an aliquot of the Ni-NTA elution fraction was reduced with DTT, alkylated with iodacetamide and then hydrolyzed with trypsin. The resulting peptides were processed by the Keck MS & Proteomics Resource Laboratory at Yale University using a Waters nano ACQUITY column (75 μm x 250 mm) followed by an Orbitrap Elite mass spectrometer. The presence of Bta instead of Trp is indicated if MASCOT finds a Trp + 17.05 Da residue at an annotated Trp position in the database (SwissProt).

### Size exclusion chromatography of thioredoxin

The molecular weight of the Trx variants has been determined by size exclusion chromatography on a Superdex 75 HR 10/30 column connected to an ÄTKA explorer (GE healthcare, Bio-Sciences, Pittsburgh, USA). 100 μl of the Ni-NTA elution fractions were loaded and eluted in gel filtration buffer (20 mM HEPES–NaOH, pH 7.7, 150 mM NaCl) at a flow rate of 0.5 ml/min.

### Enzymatic assay of thioredoxin and variants

The enzymatic activities of the Trx variants were analyzed within the sequential reduction in the order of oxidized ArsC, Trx, TrxR to NADPH/H^+^. With an excess of TrxR, the NADPH/H^+^ consumption reflects the activity of Trx depending on the oxidized ArsC concentration. Measurements were performed in the anaerobic tent using a NanoDrop device with UV-transparent ultra-micro cuvettes (USA Scientific, Ocala, USA). 100 μl reactions consist of 50 mM Tris–HCl, pH 7.6, 200 mM NaCl, 0–160 μM oxidized ArsC, 0.125 μM Trx, 4 μM TrxR and 250 μM NADPH^+^ with all components prewarmed to 37°C (except oxidized ArsC which was kept at RT to prevent precipitation). After oxidized ArsC was added, the initial velocities were measured by the decline of A_340_ through NADPH/H^+^ consumption (less than 2 min). Using the NADPH/H^+^ molar extinction coefficient *ϵ*_340_ = 6220 M^−1^ cm^−1^, the kinetic parameters of Trx variants have been calculated using the GraphPad Prism 6 software (La Jolla, USA). The plot of the reaction velocity against the substrate concentration was used for non-linear regression to obtain *k*_cat_ and *K*_M_. Standard errors were calculated by the deviations of the measured values from the fitted curve.

## RESULTS

### Engineering of PylRS variants for acylating tRNA with Bta

New PylRS variants were selected from a mutation library of *Methanosarcina mazei* PylRS containing three types of combinations of random mutations: (i) a previously described group with mutations of Leu305, Asn346 and Cys348 (20); (ii) a group with mutations of Asn346, Cys348 and Tyr306 and (iii) a group with mutations of Asn346, Cys348, Leu309, with indicated residues randomized as NNK (where N stands for A, G, C or T and K stands for G or T in the codon of the amino acid) in all three sub-libraries. Initially, the enzymes were screened for incorporation of the ncAA 3-I-Phe and then selected via three rounds of the standard positive-negative-positive selection procedure in *E. coli* TOP10 cells (20,26). The two surviving mutants were found to carry two mutations at positions Asn346 and Cys348 (Asn346Gly/Cys348Gln and Asn346Ser/Cys348Gln) which we named BtaRS and 3-methyl-PheRS (MFRS), respectively (Supplementary Table S2).

PylRS variants typically have broad ncAA substrate specificity (18,20). Therefore, we wanted to test the substrate specificity of the newly evolved PylRS variants. Using a sfGFP reporter gene with a TAG in codon position 2 (sfGFP-2TAG) (20) we determined the *in vivo* translation activities with a range of ncAAs aminoacylated by MFRS and BtaRS. Using 3-I-Phe as the substrate, both MFRS and BtaRS supported efficient UAG suppression (Table 1). MFRS and IFRS had nearly same substrate range for multiple Phe derivatives (Supplementary Figure S2) (20); this indicates that the Ile348 (IFRS) to Gln348 (MFRS) substitution has only a minor effect on the active site pocket. The BtaRS substrate range was broader (Supplementary Figure S3) and led to efficient incorporation of the Trp analog Bta, as judged by the intensity of sfGFP fluorescence (Figure 1A and Supplementary Figure S3). To confirm that the increased fluorescence reflected Bta incorporation, we purified the sfGFP-2Bta protein and confirmed Bta incorporation by determining the mass of both the intact protein and of the tryptic peptide (Figure 1B and Supplementary Figure S4). In an analogous experiment no Trp incorporation was detected, pointing to the high Bta *vs* Trp specificity of BtaRS (Figure 1A and Table 1).

**Table 1.** Apparent kinetic parameters of *M. mazei* PylRS variants for amino acid activation

Enzyme	Amino acids	K_M_ (μM × 10^3^)	*k*_cat_ (s^−1^ × 10^−2^)	*k*_cat_/*K*_M_ (μM^−1^s^−1^ × 10^−5^)	Relative catalytic efficiency	UAG translation efficiency
MmPylRS^1^	Pyl	0.05 ± 0.008	29.8 ± 1.2	596	100	15± 1.5
BtaRS	3-I-Phe	0.95 ± 0.11	8.9 ± 0.3	9.37	1.6	44 ± 5.1
	3-CF_3_-Phe	1.44 ± 0.20	11.2 ± 0.5	7.78	1.3	39 ± 4.9
	3-Br-ThA	1.13 ± 0.35	7.3 ± 0.8	6.46	1.1	2 ± 1.0
	Bta	0.37 ± 0.07	5.3 ± 0.2	14.3	2.4	38 ± 6.4
	Trp	ND^b^	ND	-	-	-
MFRS	3-I-Phe	3.18 ± 0.53	19.3 ± 1.5	6.07	1.0	47 ± 5.7
	3-CF_3_-Phe	2.16 ± 0.21	9.9 ± 0.4	4.58	0.77	41 ± 6.3
	3-Br-ThA	1.55 ± 0.34	6.9 ± 0.7	4.45	0.75	8 ± 0.9
IFRS^a^	3-I-Phe	0.82 ± 0.09	8.71 ± 0.33	10.6	1.8	48 ± 0.1
	3-CF_3_-Phe	1.13 ± 0.10	7.29 ± 0.21	6.45	1.1	53 ± 2.3
	3-Br-ThA	1.57 ± 0.32	4.43 ± 0.27	2.82	0.47	7 ± 0.9

^a^Kinetic data were adapted from previous work (20).

^b^ND represents not detectable.

### Kinetic characterization of BtaRS and MFRS

Since the ease of *in vivo* UAG translation of the *sfGFP-2TAG* gene is determined by many factors (e.g. ncAA cell permeability, chemical stability, or ncAA-tRNA compatibility with EF-Tu (27)), we characterized our evolved PylRS variants *in vitro* by measuring the activation of several ncAAs in the ATP-PP_i_ exchange reaction (20). The Bta activation by BtaRS reached 2.4% of the catalytic efficiency that PylRS displays for its cognate Pyl substrate (Table 1). To our knowledge, this is the highest observed ncAA activation by any PylRS variant evolved so far, exceeding the previously published IFRS which was best when activating 3-I-Phe (Table 1). We also observed a comparable, though lower degree of activation of other ncAAs, including 2-(5-Bromothienyl)alanine, meta substituted iodo- and trifluormethyl-phenylalanine derivatives by the three engineered PylRS variants (between 0.47 and 1.8% compared to WT PylRS and Pyl) (Table 1). At the same time, despite the high structural similarity between Bta and Trp, the latter amino acid could not be activated by BtaRS in line with the *in vivo* incorporation results.

### Structural basis of Bta discrimination from Trp and other amino acids by the engineered BtaRS

To understand how BtaRS discriminates the Bta molecule from Trp and other canonical amino acids, we determined a crystal structure of BtaRS•Bta complex. To prevent poor solubility of BtaRS, we used a C-terminally truncated variant of BtaRS. Even though this BtaRS variant was still poorly soluble, addition of Bta (to 5 mM final concentration) increased its solubility to ∼12 mg/ml, which was sufficient for crystallization. The crystal structure of BtaRS in complex with Bta and ATP was determined at a resolution of 2.1 Å (Supplementary Table S1). The unbiased *F*_o_ − *F*_c_ electron density map revealed the presence of both Bta and ATP in the active site of the enzyme (Supplementary Figure S5).

The overall structure of BtaRS is similar to those of *M. mazei* PylRS (PDB 2ZIM) and PylRS variant IFRS (PDB 4TQD), but it has two amino acid mutations in the active site pocket (Figure 1C and D) (17,18). The first mutation, Asn346Gly, creates a flat interface in the amino acid-binding pocket, which shape complements the shape of the Bta molecule (Figure 1D). The second mutation, Cys348Gln, enables an additional contact between BtaRS and Bta. Together, the mutated residues appear to help discriminate of Bta from those amino acids, which lack large hydrophobic indole-like ring. We depicted the van der Waals radius of Bta and the surrounding residues that are involved in substrate recognition (Figure 1D). Apparently, the discrimination between Bta and Trp depends on two other residues in the BtaRS active site, Val401 and Trp417 (Figure 1D). These residues form hydrophobic contacts with the Bta sulfur atom, which would be impossible for polar and hydrogen-containing indole imino group of the Trp molecule (Supplementary Figure S6). Thus, BtaRS discriminates Bta from canonical amino acids apparently by four residues in its active center – Val401 and Trp417, which are also present in the WT PylRS, and Gly346 and Gln348, which were introduced in the Bta structure by mutagenesis.

Compared to the 3-I-Phe bound in IFRS active site (20), the BtaRS•Bta complex shows that the carboxyl group of Bta shifts away from the ATP binding site, leading to an increased distance between the carboxyl oxygen of Bta and the α-phosphate of ATP. Although a water molecule (not shown in Figure 1D) mediates the interaction between the amino group of Bta and the α-phosphate of ATP, this conformation of Bta is still less reactive as shown by our kinetic measurements that the *k*_cat_ value for Bta is lower than that for 3-I-Phe (Table 1).

### Probing the role of the conserved Trp28 in *S. aureus* thioredoxin

**Figure 2.**
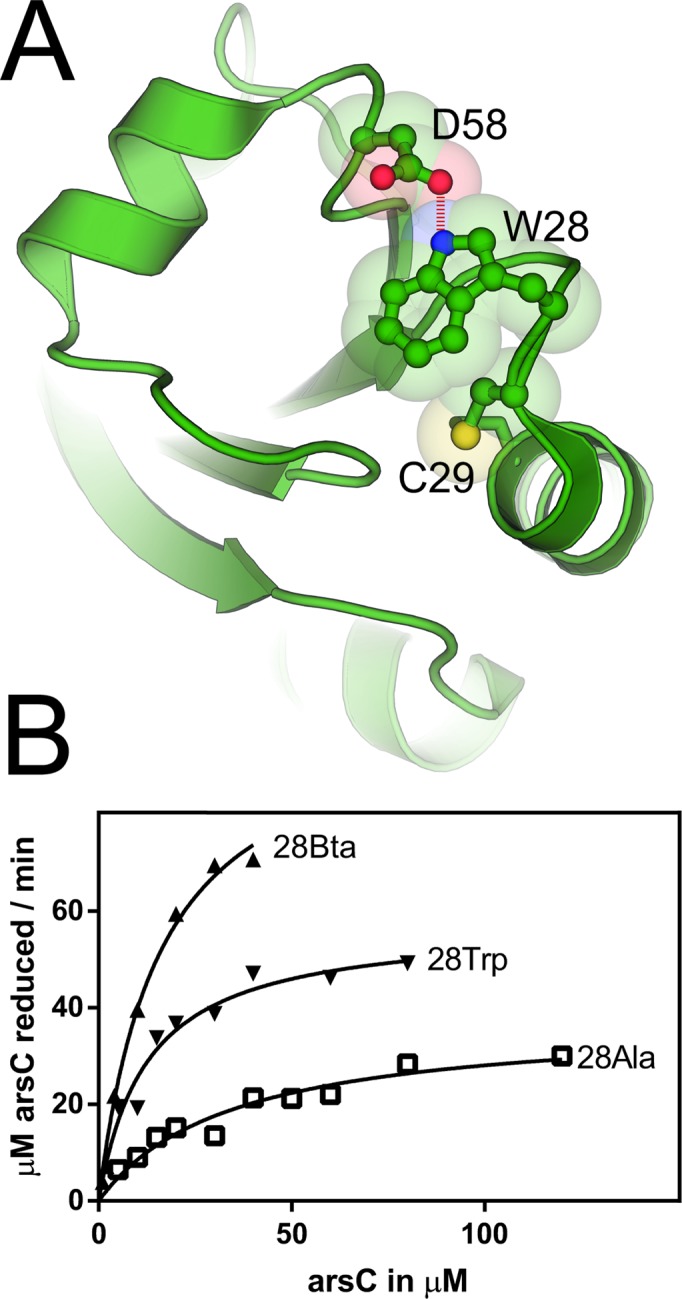
*S. aureus* Trx structure and kinetic values of indicated variants. (**A**) The Trx structure (taken from (29)) is shown in the cartoon representation with the highlighted residues. Trp28 forms an H bond with Asp58 and participates in deprotonation of Cys29 to form the active thiolate. The oxidized Trx with a Cys29—Cys32 disulfide bond is shown. (**B**) The reaction velocities of the Trx enzymes Trp28 (WT), Bta28 and Ala28 are plotted against the substrate concentration of oxidized ArsC.

To illustrate a possible application of BtaRS in protein structural and functional study, we used BtaRS to introduce an imino-to-sulfur substitution into the active site Trp of *S. aureus* Trx *in vivo*. A small, ubiquitous thiol protein Trx is one of the most important regulators of cell redox balance. Trx acts as antioxidant to reduce disulfide bonds in target proteins by catalyzing a cysteine thiol-disulfide exchange reaction. Its activity relies on two cysteines (Cys29 and Cys32 in *S. aureus* Trx) residing in the highly conserved WCGPC motif which comprises the active site of the enzyme. A biochemical study showed that *S. aureus* Trx stability and activity also critically depends on the conserved Trp28 residue located adjacent to the catalytic site (28). In the crystal structure of the *S. aureus* Trx, the indole side chain of Trp28 was found to shield the active site from solvent (Figure 2A) (29). A Trp28Ala mutation caused a ten-fold decrease of Trx activity and accumulation of inactive Trx dimers (28). Three alternative scenarios were offered to explain this effect. (i) The loss of activity is the result of an impaired hydrogen bond between Trp28 and Asp58. (ii) The different chemical structure of Ala residue causes a substantial deformation of the active site. (iii) The indole ring of Trp28 may act as a base to help deprotonation of Cys29 to form the reactive thiolate (28,29).

To shed further light on these scenarios we replaced Trp28 with Bta28, and also made the Ala28 variant. Then we measured the enzymatic activity of these variants and the WT enzyme *in vitro* (Figure 2B). For this purpose, Trx had been embedded in the reduction cascade of oxidized ArsC, Trx, TrxR and NADPH/H^+^, which allowed monitoring of the Trx activity by measuring A_340_. TrxR was present in excess so that the overall reaction was limited by Trx and depended on the varied oxidized ArsC concentration. The obtained Michaelis–Menten parameters for the WT and Ala28 enzymes (Table 2) differ from the previously published values (30). While we also obtained less active (lower *k*_kat_, higher *K*_M_) enzymes when working aerobically, the strict anaerobic work without any reducing agents abolished the interfering air oxygen and DTT completely. Both, Trx WT and 28Ala variants have 3- and 10-fold higher *k*_cat_ and about 3-fold lower *K*_M_. However, even though the Bta28 variant cannot form the H bond with Asp58, its catalytic efficiency is 37% higher than that for the WT enzyme (Table 2). As indicated above (Figure 1), Bta differs from Trp by the replacement of an imino group with a less electronegative sulfur. Hence, the higher aromatic electron density of Trx Bta28 should serve as a stronger base for C29 thiolate formation.

**Table 2.** Apparent kinetic parameters of *S. aureus* Trx and variants for ArsC reduction

Trx	*K*_M_ (μM)	*k*_cat_ (min^−1^)	*k*_cat_/*K*_M_ (μM^−1^min^−1^)	Relative catalytic efficiency	Source
WT	12 ± 3	460 ± 33	38	100	This work
Trp28Ala	43 ± 9	336 ± 28	7.8	21	This work
Trp28Bta	16 ± 3	824 ± 60	52	137	This work
WT	33	114	3.5	-	(30)
Trp28Ala	110	37.7	0.34	-	(28)

To monitor dimerization of Trx mutants, we used the size exclusion chromatography. Similar to WT Trx, Trx Bta28 eluted primarily in a monomeric form and only a minor peak for possible cysteine cross-linked dimers had been observed (Supplementary Figure S7), indicating that the loss of the hydrogen bond to Asp58 does not have a severe impact on the structural fold and most of the enzyme stayed in the monomeric and active form.

## DISCUSSION

Noncanonical amino acids are not only used for introduction of reactive handles allowing coupling with fluorescent dyes, ligands or proteins (reviewed in (31)), but also co-translational incorporation of residues resembling post-translational modifications (N-acetyl-lysine (26,32) and phosphoserine (27)) allowed studying proteins in their activated forms. To probe the importance of an active site residue, the effect of its mutation to an alanine residue is mostly employed. However, some residues (Tyr and Trp) harbor several functional groups, making a dissection of individual contributions from each functional group challenging. Recently, the active site Tyr residue of myoglobin has been replaced by 2,3,5-trifluorotyrosine; lowering the p*K*_a_ of the phenolic hydroxyl group from 10.0 to 6.4 led to a doubling of the oxidase activity (33). The voltage-gated potassium (Kv) channel in frog oocytes has been studied in a mutagenic analysis. Mutating Trp434 to Tyr or Phe perturbed the local structure and resulted in fast inactivation, while the H-bond deficient Bta resulted in slower channel inactivation compared to the WT protein (34). For this purpose, an amber suppressor tRNA was chemically acylated with Bta and then microinjected into frog oocytes.

The BtaRS evolved in this work is based on the PylRS•tRNA^Pyl^ pair which has been shown to be orthogonal in *E. coli*, yeast, and mammalian cells. While an application of genetic code expansion with Bta in *E. coli* has been shown here, the system should be adaptable to mammalian cell culture, if Bta can permeate the mammalian cell wall.

The BtaRS•Bta structure suggests how BtaRS discriminates Bta from canonical amino acids. Relative to PylRS, BtaRS carries two mutations in the amino acid binding site – Asn346Gly and Cys348Gln. Both these mutated residues form a binding site for the Bta molecule (Figure 1D). It appears from the BtaRS•Bta interface that these mutated residues reprogram PylRS specificity to recognize large and flat aromatic structures, such as the aromatic rings of Bta or Trp. Importantly, two conserved residues in the BtaRS structure, Val401 and Trp417, bind the Bta sulfur atom – a contact which would not be possible for the imino group in the indole ring of Trp. Thus, the BtaRS residues Gly346 and Gln348 help create the binding pocket, which favors binding of the unique indole-like ring structures, whereas Val401 and Trp417 appear to discriminate Bta from Trp.

The application of Bta shed further light on the role of Trp28 on the activity of *S. aureus* Trx by exploiting its stronger aromatic π electron density. Our and previous data suggest that the formation of the C29 active thiolate limits the Trx reactivity, as probed with the adjacent Ala28 and non-standard Bta28 residues (35). A similar situation is seen for selenocysteine (Sec, U), the 21st genetically encoded aa - for which the Cys thiol group is replaced by the selenol group. As Sec is already deprotonated under physiological conditions (pKa 5.7), no nearby assisting residues are needed for selenolate formation. Several bacteria encode Sec-containing Trx with a (W/G)UXXC active site motif (36). However, for the *Treponema denticola* Trx similar catalytic efficiencies have been obtained for the natural Trx selenoenzyme and its Cys-substituted homolog (37).

BtaRS, evolved in this study, provides an alternative tool for genetic encoding of Bta and chemical group substitution of Trp with its hydrogen-bond deficient analog. Earlier, two enzymes were evolved for the Bta acylation (13–15). However, both these enzymes were derived from yeast aaRSs; therefore their use *in vivo* is limited to bacteria. By contrast, PylRS-derived enzymes are orthogonal and charge many ncAAs not only in bacteria, but also in many eukaryotes (18,19). Thus the key advantage of BtaRS over analogous enzymes is that BtaRS can be potentially used in a substantially broader range of species, including mammalian cells. We also determined a high resolution crystal structure of BtaRS in complex with Bta, which provides insight into the substrate recognition by PylRS, and enriches the crystal structure family of PylRS with another ncAA.

## ACCESSION NUMBER

Protein coordinates and structure factors have been submitted to the Protein Data Bank under the accession code 4ZIB.

## Supplementary Material

SUPPLEMENTARY DATA
