# Supplementary material for: Probing the active site tryptophan of Staphylococcus aureus thioredoxin with an analog
Source: Nucleic Acids Res. 2015 Nov 17;43(22):11061–7. doi: 10.1093/nar/gkv1255 (PMC4678829; doi:10.1093/nar/gkv1255)
Supplement: SUPPLEMENTARY DATA [file supp_43_22_11061__index.html]

Probing the active site tryptophan of Staphylococcus aureus thioredoxin with an analog — Probing the active site tryptophan of Staphylococcus aureus thioredoxin with an analog — SUPPLEMENTARY DATA 

# Probing the active site tryptophan of *Staphylococcus aureus* thioredoxin with an analog

## SUPPLEMENTARY DATA

- SUPPLEMENTARY DATA
