# Supplementary material for: Probing the active site tryptophan of Staphylococcus aureus thioredoxin with an analog
Source: Nucleic Acids Res. 2015 Nov 17;43(22):11061–7. doi: 10.1093/nar/gkv1255 (PMC4678829; doi:10.1093/nar/gkv1255)
Supplement: SUPPLEMENTARY DATA [file supp_gkv1255_nar-02222-y-2015-File004.pdf]

Table S1. Data collection and refinement statistics

	BtaRS•Bta
**Data collection**	
Wavelength (Å)	0.9789
Space group	$P6_4$
Cell dimensions	
a, *b*, *c* (Å)	105.14, 105.14, 70.92
Resolution (Å)	42.23-2.05 (2.18-2.05)
R_{merge}	6.2 (63.4)
$I / \sigma I$	31.2 (4.75)
Completeness (%)	99.4 (99.2)
Redundancy	19.7 (20.2)
**Refinement**	
Resolution (Å)	42.23-2.05
No. reflections	27798
R_{work} / R_{free}	17.0 / 19.8
No. atoms	2353
Protein	2137
Ligand/ion	77
Water	139
B-factors	
Protein	48.50
Ligand/ion	49.50
Water	50.70
R.m.s. deviations	
Bond lengths (Å)	0.003
Bond angles (°)	0.73

[a]Values in parentheses are for highest-resolution shell. Each data set was collected from a single crystal.

Table S2. Sequence comparison of three *M. mazei* PylRS variants[1]

PylRS	N346	C348	Source
IFRS	S	I	(1)
MFRS[2,3]	S	Q	This work
BtaRS[3]	G	Q	This work

[1]The tRNA used in this work originates from *M. mazei* with the sequence of GGAAACCUGAUCAUGUAGAUCGAAUGGACUCUAAAUC
CGUUCAGCCGGGUUAGAUUCCCGGGGUUUCCGCCA

[2]MFRS has an additional mutation: V169I

[3]The amino acid sequences of MFRS and BtaRS are listed below:

MFRS: MDKKPLNTLISATGLWMSRTGTIHKIKHHEVSRSKIYIEMACGDHLVVNNSRSSRTARALRHHKYRKTCKRCRVSDEDLNKFLTK
ANEDQTSVKVKVVSAPTRTKKAMPKSVARAPKPLENTEAAQAQPSGSKFSPAIPVSTQESVSVPASVSTSISSISTGATASALIKGNTNPIT
SMSAPVQASAPALTKSQTDRLEVLLNPKDEISLNSGKPFRELESELLSRRKKDLQQIYAEERENYLGKLEREITRFFVDRGFLEIKSPILIPL
EYIERMGIDNDTELSKQIFRVDKNFCLRPMLAPNLYNYLRKLDRALPDPIKIFEIGPCYRKESDGKEHLEEFTMLSFQQMGSGCTRENLES
IITDFLNHLGIDFKIVGDSCMVYGDTLDVMHGDLELSSAVVGPIPLDREWGIDKPWIGAGFGLERLLKVKHDFKNIKRAARSESYYNGISTN
L

BtaRS: MDKKPLNTLISATGLWMSRTGTIHKIKHHEVSRSKIYIEMACGDHLVVNNSRSSRTARALRHHKYRKTCKRCRVSDEDLNKFLTK
ANEDQTSVKVKVVSAPTRTKKAMPKSVARAPKPLENTEAAQAQPSGSKFSPAIPVSTQESVSVPASVSTSISSISTGATASALVKGNTNPI
TSMSAPVQASAPALTKSQTDRLEVLLNPKDEISLNSGKPFRELESELLSRRKKDLQQIYAEERENYLGKLEREITRFFVDRGFLEIKSPILIP
LEYIERMGIDNDTELSKQIFRVDKNFCLRPMLAPNLYNYLRKLDRALPDPIKIFEIGPCYRKESDGKEHLEEFTMLGFQQMGSGCTRENLE
SIITDFLNHLGIDFKIVGDSCMVYGDTLDVMHGDLELSSAVVGPIPLDREWGIDKPWIGAGFGLERLLKVKHDFKNIKRAARSESYYNGIST
NL

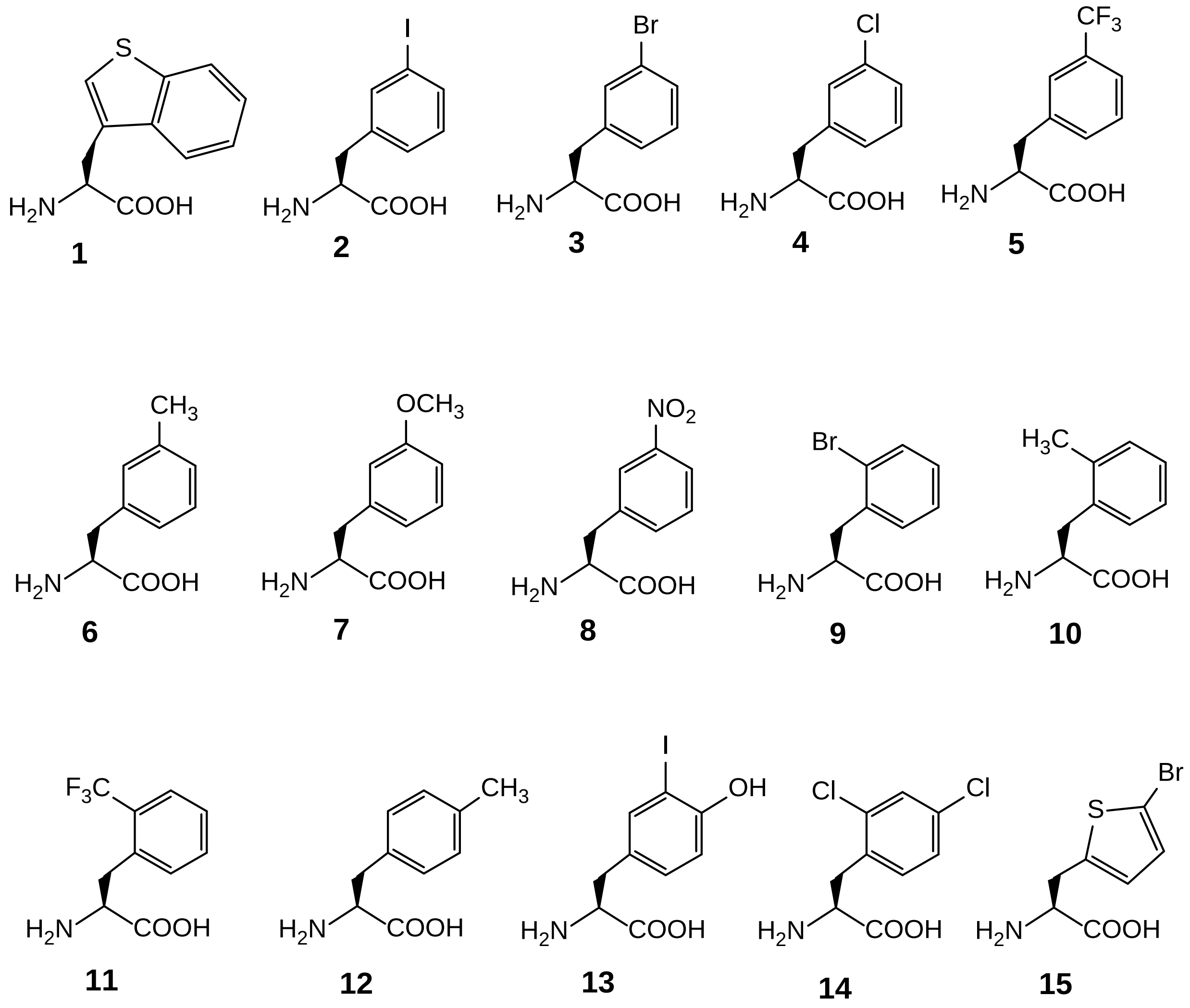

Scheme S1. Chemical structures of ncAAs used in the study.

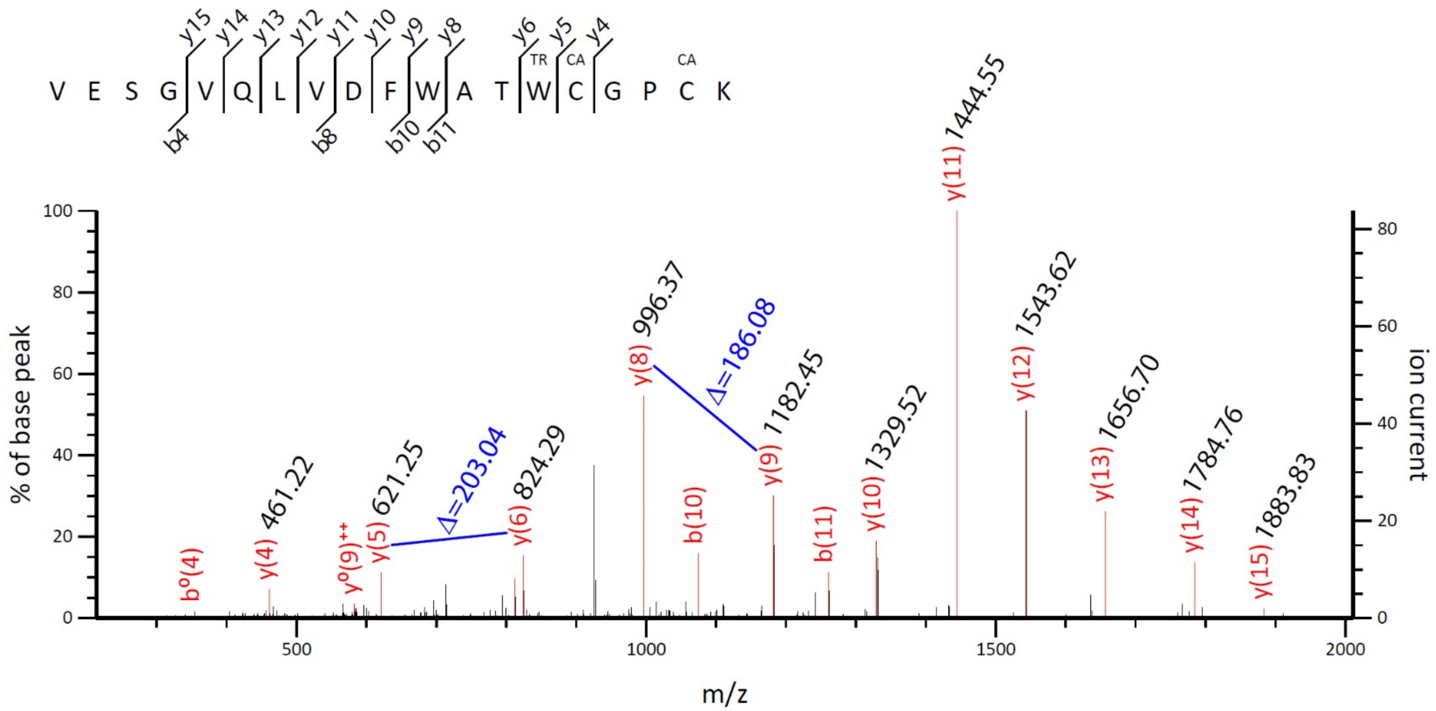

Figure S1. Tandem mass spectra of the VESGVQLVDFWATW*CGPCK (W* denotes Bta) fragment with carbamidomethyl modifications of Cys29 and Cys32 from the full-length Trx protein.

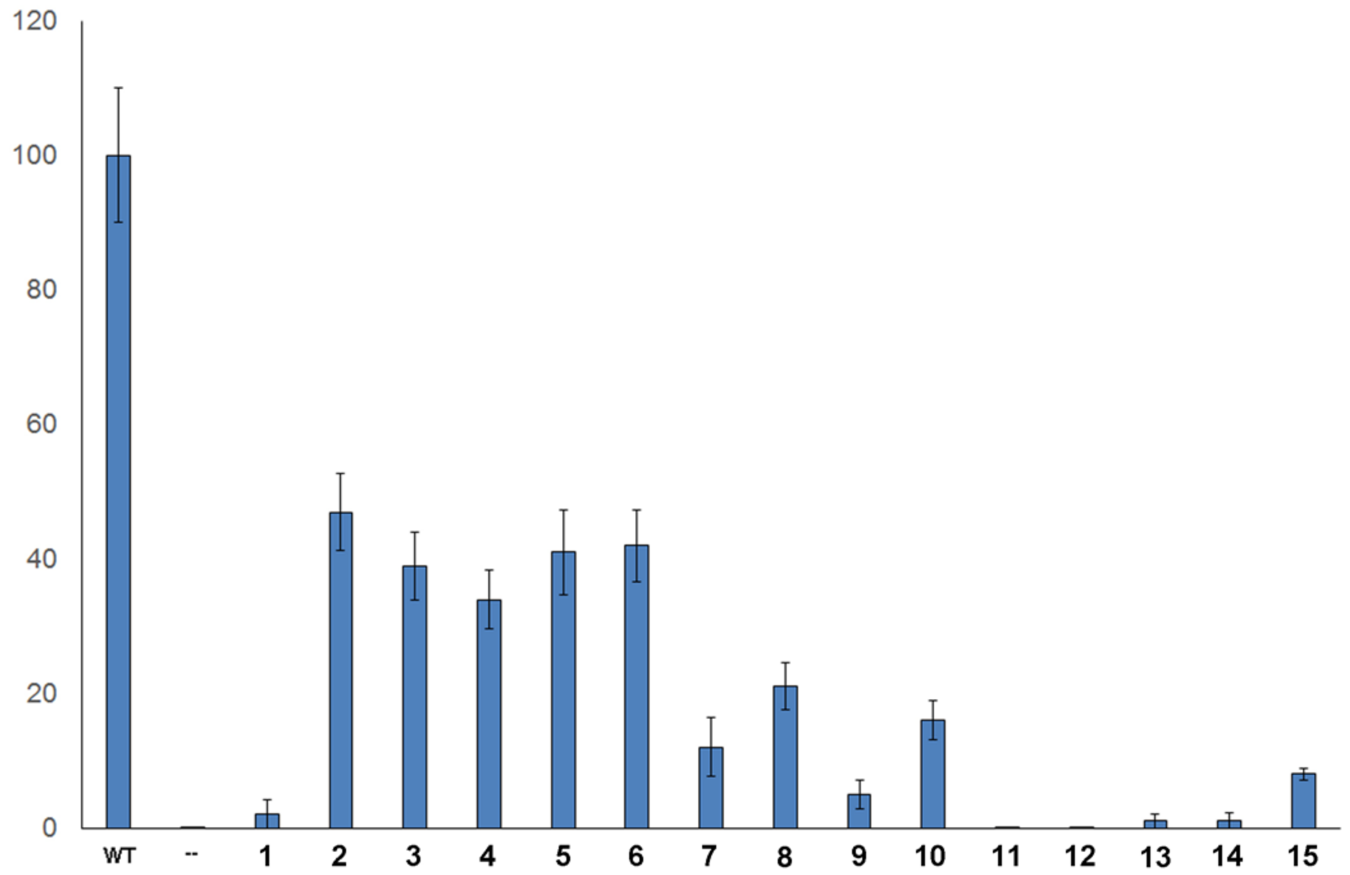

Figure S2. Substrate specificity range of MFRS•tRNA$^{Pyl}_{CUA}$. Suppression of the *sfGFP-UAG2* gene by a library of 94 ncAA-tRNAs was investigated by measuring their fluorescence intensities. Here 15 ncAAs were shown for co-translational incorporation. The first column was a positive control experiment to detect production signal of wild type *sfGFP* (100%). The second column was control experiment, lacking ncAA, to determine the background signal, which was subtracted from all experiments. The *E. coli* cell strain BL21 (DE3) was used in all of these experiments. The 94 ncAA chemical names and structures has been published previously (1). The 15 ncAA chemical structures are given in Scheme S1. Fluorescence data and error values (standard deviation) are represented by bars from three independent experiments.

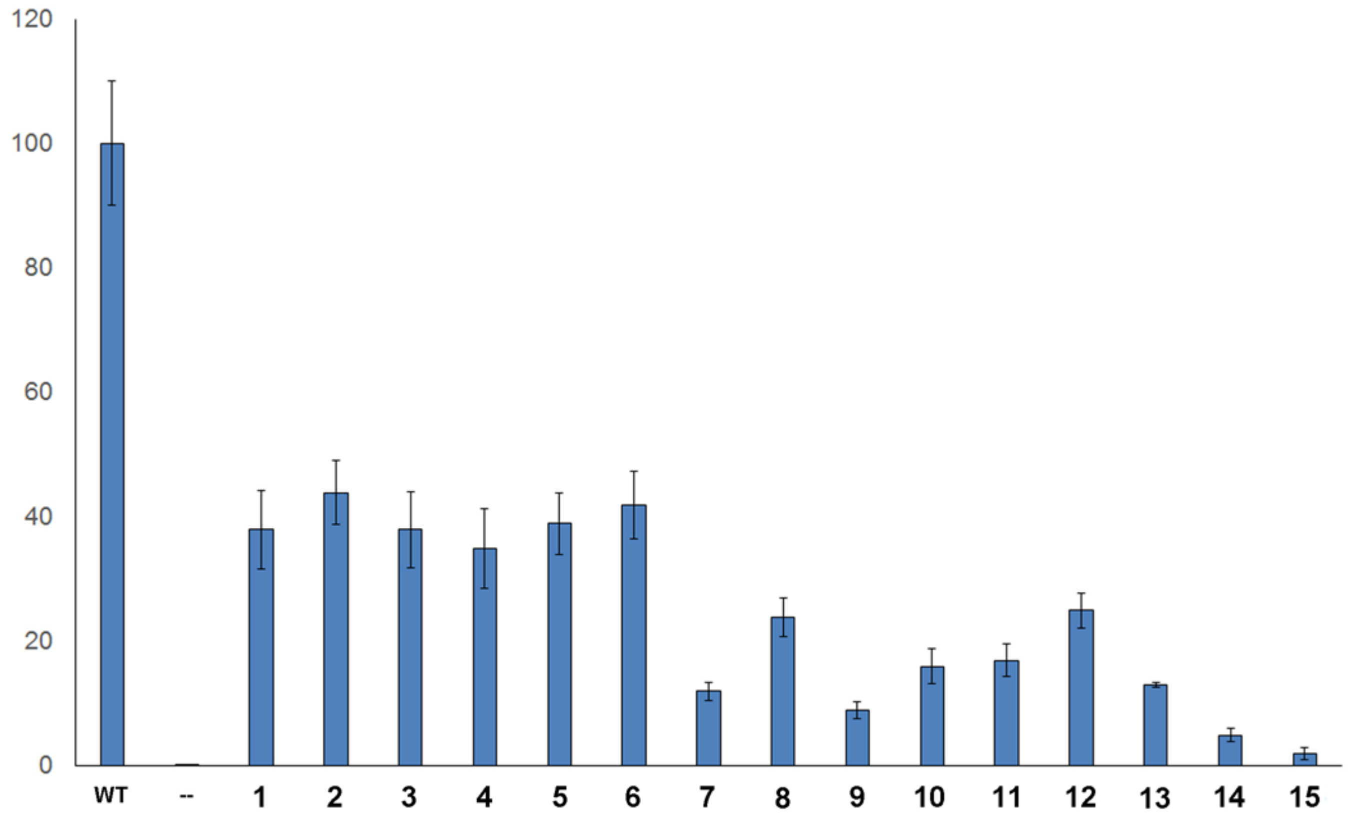

Figure S3. Substrate specificity range of BtaRS•tRNA$^{Pyl}_{CUA}$. Suppression of the sfGFP-UAG2 gene by a library of 94 ncAA-tRNAs was investigated by measuring their fluorescence intensities. 15 ncAAs were shown for co-translational incorporation. The first column was a positive control experiment to detect production signal of wild type sfGFP (100%). The second column was control experiment, lacking ncAA, to determine the background signal, which was subtracted from all experiments. The *E. coli* cell strain BL21 (DE3) was used in all of these experiments. The 94 ncAA chemical names and structures has been published previously (1). The 15 ncAA chemical structures are given in Scheme S1. Fluorescence data and error values (standard deviation) are represented by bars from four independent experiments. Comparing with substrate specificity range of MFRS•tRNA$^{Pyl}_{CUA}$, ncAA screening of of BtaRS•tRNA$^{Pyl}_{CUA}$ pair shows additional ncAA responses **1**, **11-14**.

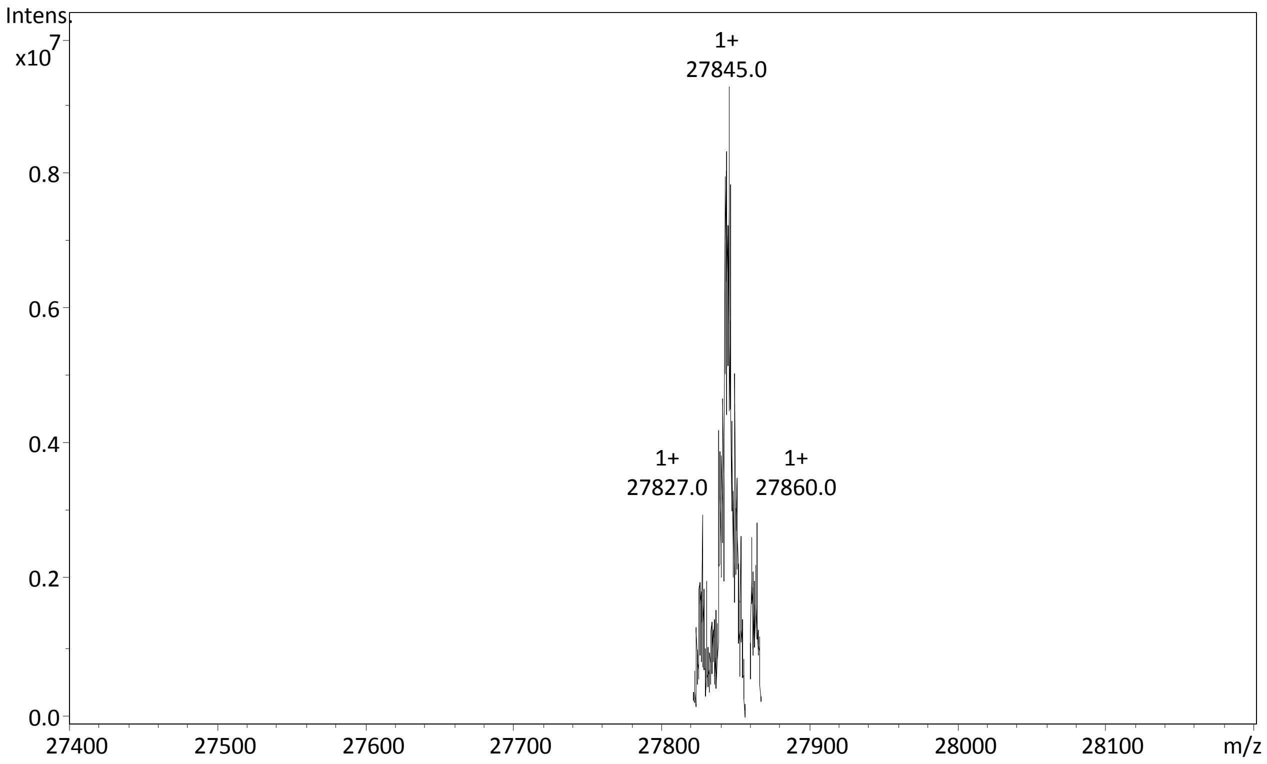

Figure S4. Molecular weight determination of the protein sfGFP-Bta. The deconvoluted singly charged ESI-MS spectrum of sfGFP-Bta by FT-ICR MS is shown. The full-length sfGFP-Bta protein was expressed using the BtaRS-$tRNA^{Pyl}_{CUA}$ pair in the presence of 1 mM Bta. Calculated molecular weight of full-length sfGFP-Bta protein is 27,845 Da ($[M+H]^{1+}$); we found 27,845 Da, 27,827 Da (deamidation), and 27,860 Da (Oxidation).

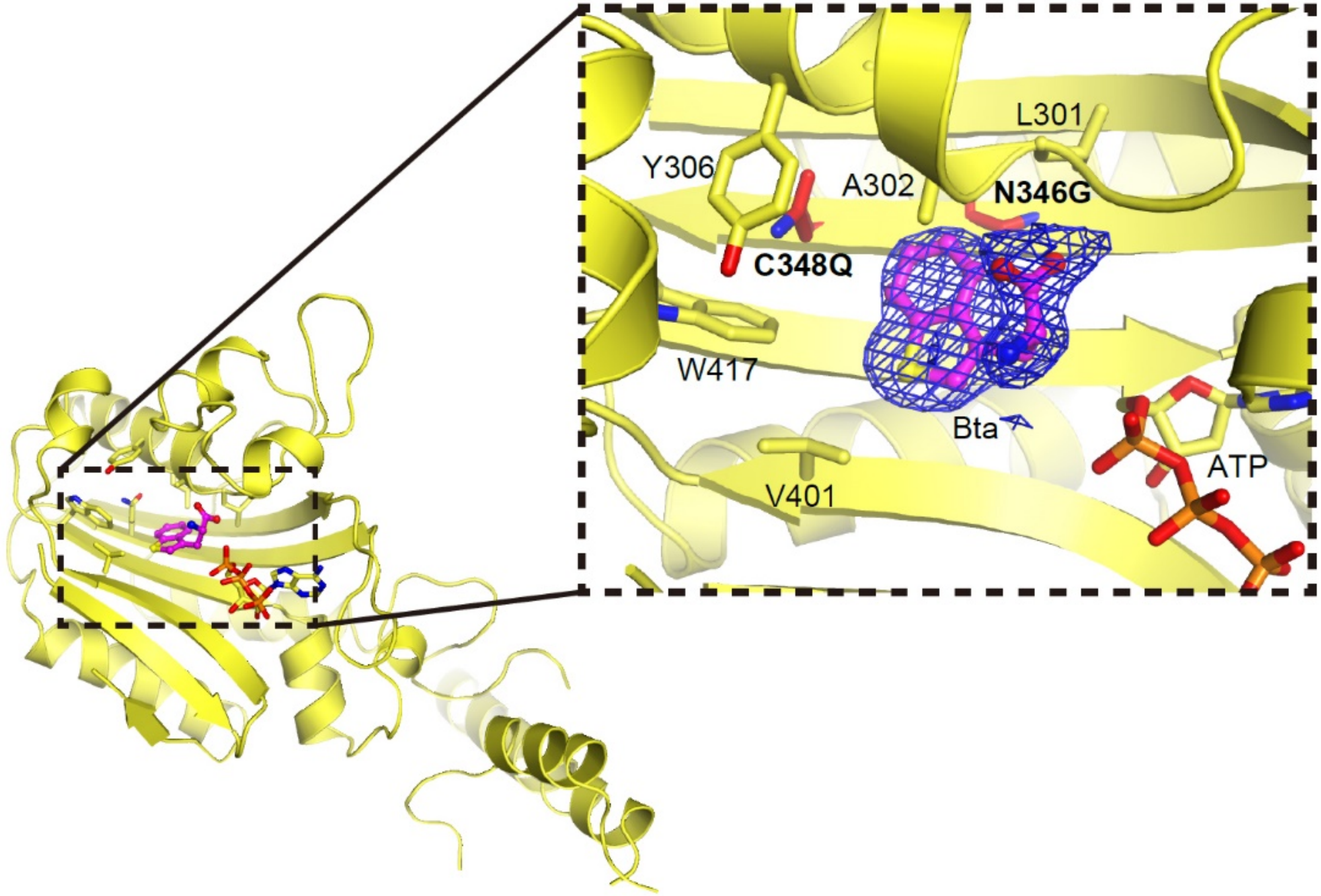

Figure S5. Complex structure of BtaRS with Bta and ATP (PDB ID 4ZIB). Close-up view of the active site are displayed, showing the residual Fourier difference (mF_o-dF_c) electron density map in blue, contoured at the 2.0σ level.

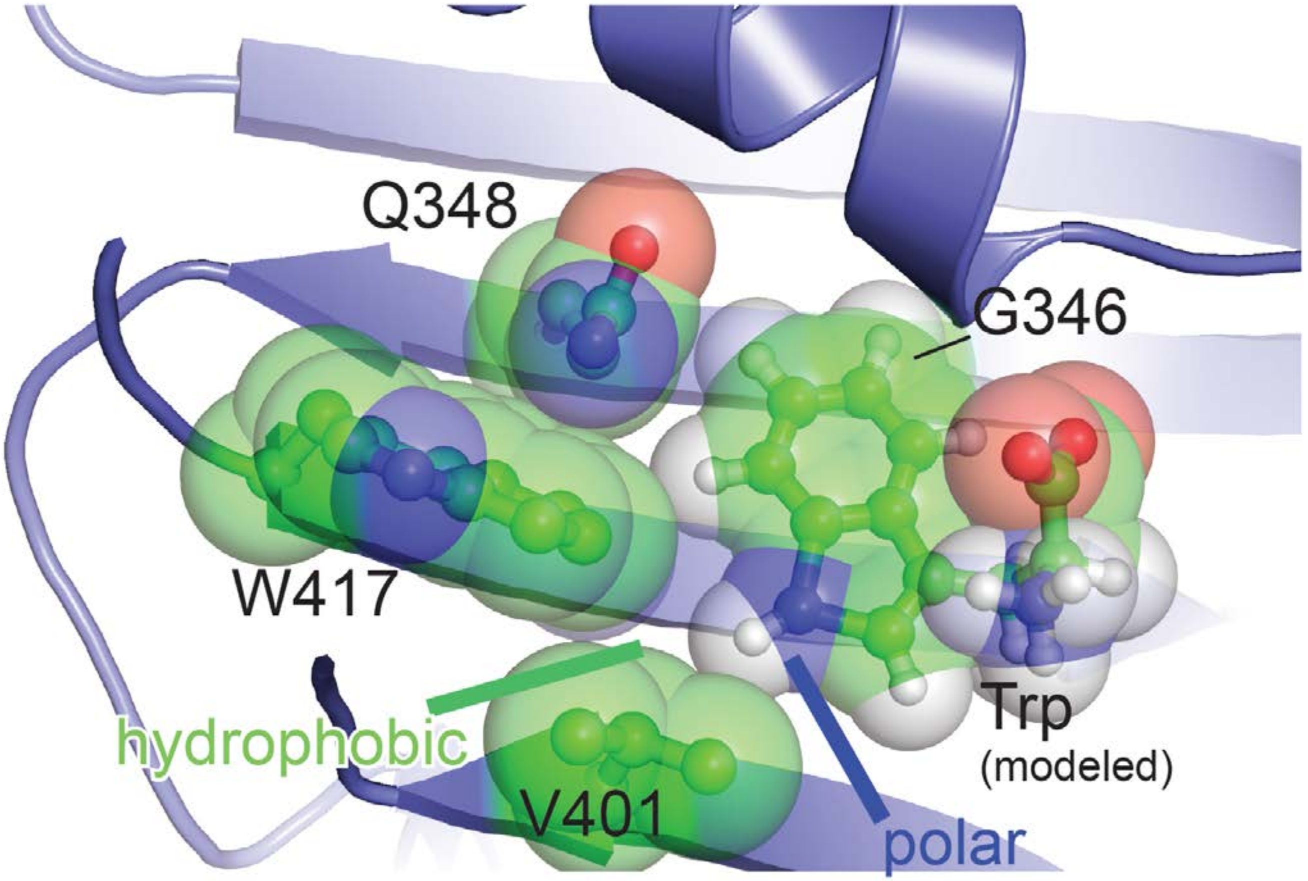

Figure S6. Complex structure of BtaRS with modeled Trp. The coordinates of Bta have been edited in UCSF Chimera to replace the sulfur with the polar imino goup which would interfere with the hydrophobic parts of the two active site residues, V401 and W417.

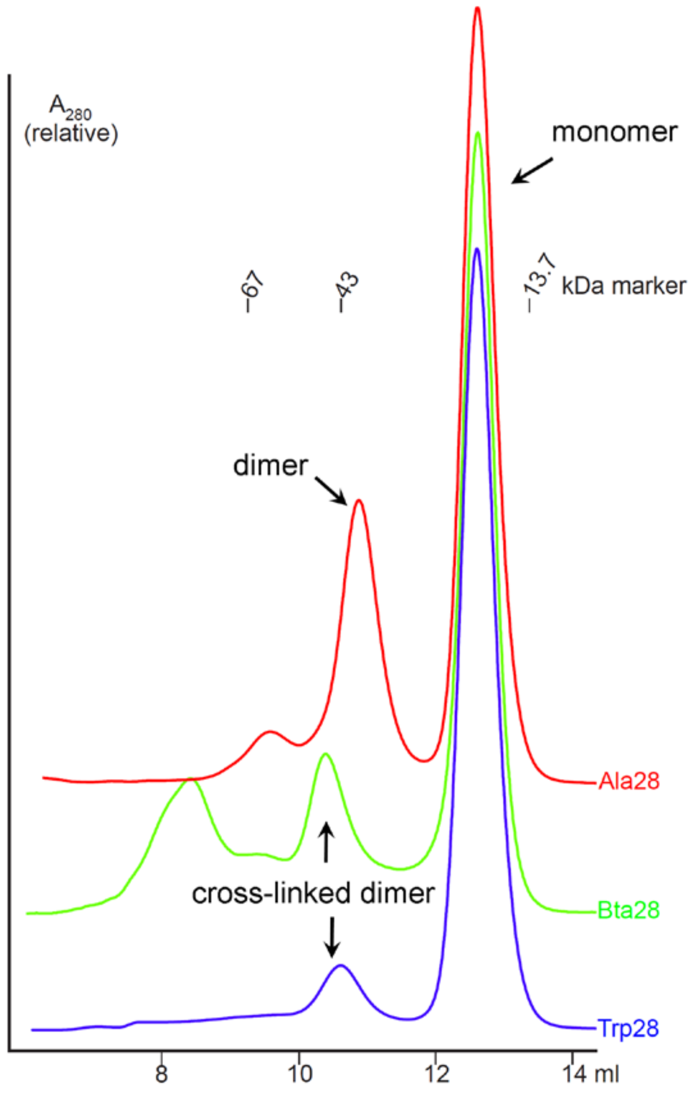

Figure S7. Size exclusion chromatography for Trx. The major peaks indicate the elution of the monomeric form of the three proteins. The dimeric form of W28A mutant and crosslinked dimer of wild-type Trx and W28Bta variant were eluted as minor peaks.

REFERENCE

1. Guo, L.T., Wang, Y.S., Nakamura, A., Eiler, D., Kavran, J.M., Wong, M., Kiessling, L.L., Steitz, T.A., O'Donoghue, P. and Söll, D. (2014) Polyspecific pyrrolysyl-tRNA synthetases from directed evolution. *Proc. Natl. Acad. Sci. USA*, **111**, 16724-16729.
